# Characterization of Pure Ductal Carcinoma In Situ on Dynamic Contrast-Enhanced MR Imaging: Do Nonhigh Grade and High Grade Show Different Imaging Features?

**DOI:** 10.1155/2010/431341

**Published:** 2010-09-21

**Authors:** Siwa Chan, Jeon-Hor Chen, Garima Agrawal, Muqing Lin, Rita S. Mehta, Philip M. Carpenter, Orhan Nalcioglu, Min-Ying Su

**Affiliations:** ^1^Department of Radiology, Taichung Veterans General Hospital, Taichung 404, Taiwan; ^2^Tu and Yuen Center for Functional Onco-Imaging and Department of Radiological Science, University of California, Irvine, Ca 92697, USA; ^3^Department of Radiology, China Medical University Hospital, Taichung 404, Taiwan; ^4^Department of Medicine, University of California, Irvine, Ca 92868, USA; ^5^Department of Pathology, University of California, Irvine, Ca 92868, USA

## Abstract

To characterize imaging features of pure DCIS on dynamic contrast-enhanced MR imaging (DCE-MRI), 31 consecutive patients (37-81 years old, mean 56), including 2 Grade I, 16 Grade II, and 13 Grade III, were studied. MR images were reviewed retrospectively and the morphological appearances and kinetic features of breast lesions were categorized according to the ACR BI-RADS breast MRI lexicon. DCE-MRI was a sensitive imaging modality in detecting pure DCIS. MR imaging showed enhancing lesions in 29/31 (94%) cases. Pure DCIS appeared as mass type or non-mass lesions on MRI with nearly equal frequency. The 29 MR detected lesions include 15 mass lesions (52%), and 14 lesions showing non-mass-like lesions (48%). For the mass lesions, the most frequent presentations were irregular shape (50%), irregular margin (50%) and heterogeneous enhancement (67%). For the non-mass-like lesions, the clumped internal enhancement pattern was the dominate feature, seen in 9/14 cases (64%). Regarding enhancement kinetic curve, 21/29 (78%) lesions showed suspicious malignant type kinetics. No significant difference was found in morphology (*P* > .05), tumor size (*P* = 0.21), and kinetic characteristics (*P* = .38) between non-high grade (I+II) and high-grade (III) pure DCIS.

## 1. Introduction

Ductal Carcinoma In Situ (DCIS) is characterized by proliferation of malignant cells confined by the basement membranes of ductal structures, without evidence of extraductal invasion [1]. DCIS comprises different subtypes with heterogeneous proliferative disease processes and varies in architecture, imaging features, and clinical outcome. Although the progression of DCIS is not fully understood, it is known that untreated DCIS is likely to progress to invasive cancer within 10 years of diagnosis. Therefore, DCIS is considered a preinvasive form of invasive breast cancer that requires immediate treatment [2, 3].

With the widespread use of screening mammography, DCIS now accounts for 15%–20% of all newly detected breast cancer, with the trend still increasing [4]. In mammography, DCIS most frequently presents as microcalcifications, accounting for about 85%–90% of diagnosed cases. An accurate preoperative staging is very important for treatment planning of DCIS. Although mammography has been the mainstay for diagnosis of DCIS, it has limitations in defining the extension and the margin, especially in patients without microcalcifications or in those with dense breasts or breast implants. Furthermore, there is a tendency to underestimate the tumor size of DCIS on mammography [5–7]. Positive surgical margin is known as an independent risk factor for local recurrence [8–10]. 

Dynamic contrast-enhanced MRI (DCE-MRI) has been proven more sensitive than mammography for detecting breast cancer in young women with a high risk of developing breast cancer [11–13]. In many instances, DCE-MRI can reveal early stage breast cancer, including DCIS and DCIS with small invasive carcinomas, which are mammographically, sonographically, and clinically occult. In addition to screening, breast MR can also be used for staging purposes to better characterize the disease extent and the presence of multifocal multicentric lesions, which can impact on treatment planning and the subsequent management. 

Imaging characterization of patients with mixed cohort of pure DCIS and DCIS with invasive components [14–17] and patients with merely pure DCIS [18–25] on DCE-MRI has been reported before. In a study of 167 patients, Kuhl et al. found that mammography only diagnosed 56% pure DCIS preoperatively, while MRI achieved a diagnostic rate of 92%. For 89 high-grade lesions, MRI detected 87 lesions (98%) while mammography only diagnosed 52% of lesions [23]. Nevertheless, consensus regarding how MRI can be used to aid in management of pure DCIS has not been reached yet. Given the recent evidence that additional diseases diagnosed by preoperative MRI often led to more aggressive treatment yet did not improve the treatment outcome [26, 27], continuing exploration of the role of breast MRI for the diagnosis of DCIS is needed. 

The purpose of this retrospective study was to characterize the morphology and enhancement kinetic pattern of pure DCIS on DCE-MRI. Since high-grade DCIS is associated with a greater risk of local recurrence and progression to invasive breast cancer [28–30], it would be very helpful if the high-grade and low-grade DCIS could be differentiated on imaging. In this paper, the MRI features of DCIS between high-grade (Grade III) and non-high-grade (including low Grade I and intermediate Grade II) DCIS based on the van Nuys classification [31] were compared.

## 2. Materials and Methods

### 2.1. Patients

A total of 34 consecutive patients with histological-proven pure DCIS (without any microinvasion) were identified from our breast MRI database collected from 2002 to 2006. These patients came to our center to participate in a breast MRI research study due to suspicious lesions found in mammography, sonography, or physical examination. The medical records, breast MRI images, and mammogram reports (if available) were reviewed retrospectively. Among these 34 patients, three patients had received surgical excision biopsy before MR imaging, and they were excluded in the analysis. The remaining 31 patients were 37–81 years old (mean 56). The final diagnosis of pure DCIS was obtained from pathological examination of mastectomy (*N* = 21) and lumpectomy (*N* = 10) specimens. Screening mammography reports were available for 22 patients. This study was approved by the institutional review board, and informed consents were obtained from all patients.

### 2.2. MR Imaging Protocol

The MRI study was performed using a 1.5 T Phillips Eclipse MR scanner with a standard bilateral breast coil (Philips Medical Systems, Cleveland, Ohio). The imaging protocol consisted of high-resolution precontrast imaging and dynamic contrast-enhanced imaging. After setting the IV line, the patient was placed into the scanner in the prone position. The breasts were gently cushioned inside the coil to reduce motion. The localizer scan was first taken to define the location of breasts. Then a sagittal view unilateral T1-weighted precontrast images were acquired from the breast of concern, using a spin echo pulse sequence with TR = 1000 ms, TE = 12 ms, and FOV = 20 cm, and matrix size = 256 × 256. Following this, a 3D SPGR (RF-FAST) pulse sequence with 16 frames (repetitions) was prescribed for bilateral dynamic imaging. Thirty-two axial slices with 4 mm thickness were used to cover both breasts. The imaging parameters were TR = 8.1 ms, TE = 4.0 ms, flip angle = 20°, matrix size = 256 × 128, FOV = 32–38 cm. The scan time was 42 sec per acquisition. The sequence was repeated 16 times for dynamic acquisitions, four precontrast, and 12 postcontrast sets. The contrast agent (Omniscan, 1 cc/10 lbs body weight) was manually injected at the beginning of the 5th acquisition and was timed to finish in 12 seconds to make the bolus length consistent for all patients. Immediately following the contrast, 10 cc saline was injected to flush in all contrast medium.

### 2.3. Enhancement Kinetic Time Course

All images were transferred to a PC for analysis. The subtraction images at 1-minute postinjection were generated by subtracting the precontrast images acquired in Frame number 3 from the postcontrast-enhanced images acquired in Frame number 6. The maximum intensity projections (MIPs) were also generated from the subtraction images to help identify the lesion. The enhancement kinetic was analyzed from manually selected ROI (Region of Interest) based on the subtraction images at 1-minute post-injection. The enhanced tumor area was outlined on each imaging slice covering the lesion by an experienced breast radiologist; then a mean signal intensity time course from all 16 time frames was obtained. The percent enhancement time course was calculated by first subtracting the mean precontrast signal intensity (mean of first 4 frames) from each of the subsequent 12 postcontrast signal intensities and then normalized by the mean precontrast signal intensity ×100%.

### 2.4. MR Imaging Feature Analysis

Morphological appearances and enhancement kinetic features of lesions shown on MRI were categorized according to the ACR Breast Imaging Reporting and Data System (BI-RADS) breast MRI lexicon [30]. The features were evaluated by 2 radiologists (with 4 and 5 years of experience in reading breast MRI) separately. For cases with different results, they were discussed to reach a consensus. Based on the enhancement of lesions, the morphology was first separated into mass lesions and Nonmass-like lesions. Based on the lesion size, the mass type can be further separated into single focus/multiple foci (<5 mm) and mass (≥5 mm). Then for mass lesions other characteristics such as shape, margin, and internal enhancement patterns were evaluated. The Nonmass-like lesions were described by the type of enhancements (diffuse, regional, segmental, focal, ductal, linear) and the internal enhancement patterns (punctate/stippled, clumped, and heterogeneous). The tumor size was measured as the longest diameter on the maximum intensity projection (MIP) of subtraction image.

The enhancement kinetics was divided into two phases: the initial enhancement phase, defined as enhancement patterns within the first 2 minutes or before the curve starts to change, and the delayed phase, defined as enhancement pattern after 2 minutes or after the curve starts to change. The initial enhancement phase was classified as fast, medium, and slow. The delayed enhancement phase was described as persistent, plateau, and washout pattern. When the enhancement kinetics showed a rapid initial enhancement followed by washout or reaching to a plateau in the delayed phase, it was determined as suspicious of malignancy.

### 2.5. Histopathology

All cases are pure DCIS without presence of microinvasion. The pathological diagnosis was evaluated based on the van Nuys system [29]. DCIS was classified according to the nuclear grade (non-high grade versus high grade) and the morphologic subtype (comedo and noncomedo). Grade-I (low grade) and Grade-II (intermediate grade) were categorized as non-high grade, and grade-III was the high-grade.

### 2.6. Statistical Analysis

For statistical analysis, Fisher's exact test was used to examine the significant difference between non-high grade DCIS and high grade DCIS. A *P*-value < .05 was considered statistically significant.

## 3. Results

### 3.1. Patient and Tumor Characteristics

According to the nuclear grading system, 2 patients had Grade-I, 16 patients had Grade-II, and 13 patients had Grade-III, all together 18 non-high grade and 13 high grade. The mean age for non-high grade DCIS was 52 years old and 57 years old for high-grade DCIS (*P* = .27). The comedo type was noted in 12 patients (7 high grade and 5 non-high grade), and the noncomedo type was noted in 19 patients (6 high grade and 13 non-high grade) (Table 1). There was no significant difference in the histological comedo or noncomedo subtype between the non-high grade and high grade DCIS (*P* > .05).

### 3.2. MR Morphology Analysis

#### 3.2.1. Tumor Size and Mass versus Nonmass Morphology

**Figure 1:**
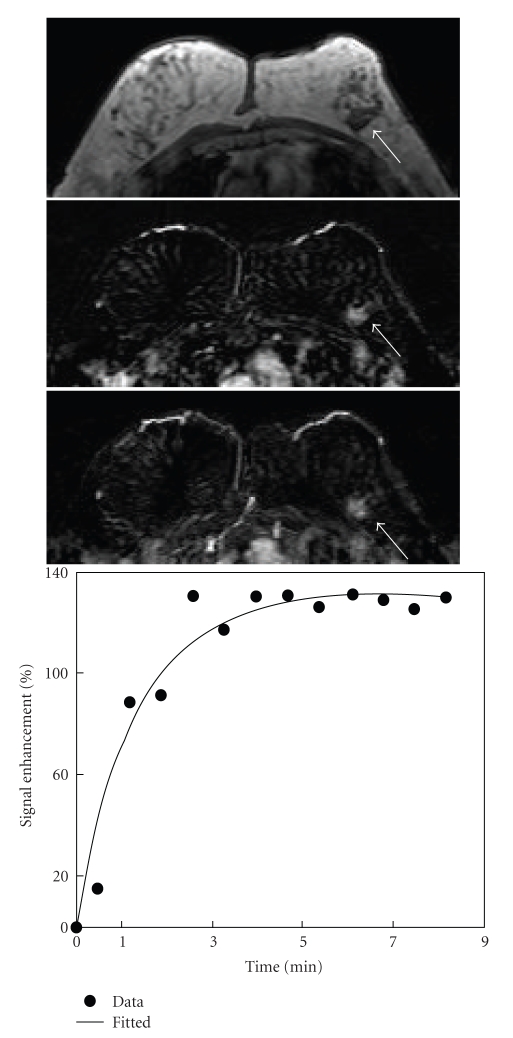
A 61-year-old woman with low-grade pure DCIS. Axial precontrast T1-weighted image (top), postcontrast subtraction image (middle), and maximum intensity projection (bottom) show an irregular mass lesion of 1.8 cm in the left breast (arrow). The enhancement level was moderate, but clearly visible. Enhancement kinetics demonstrates a moderate initial enhancement reaching to a plateau.

**Figure 2:**
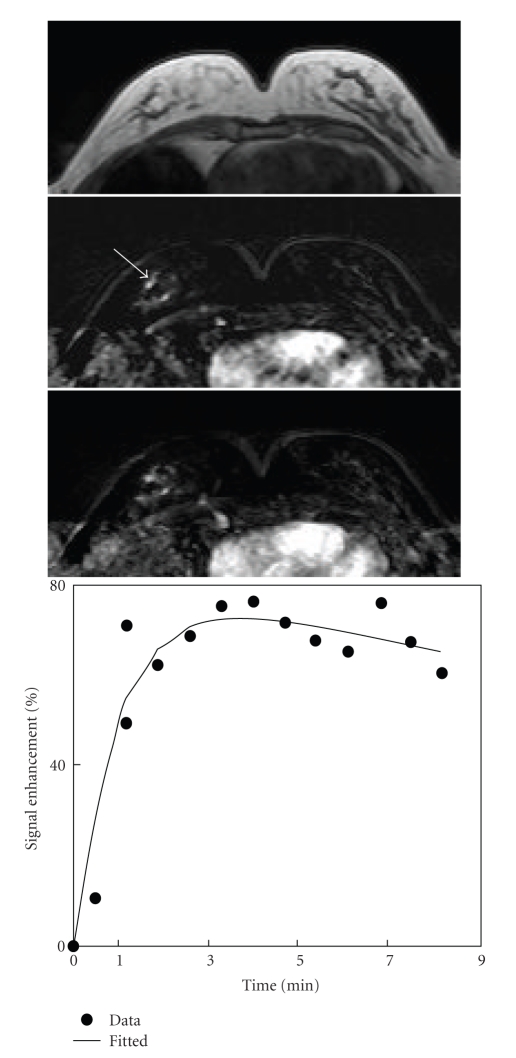
A 55-year-old woman with intermediate-grade pure DCIS. Axial precontrast T1-weighted image (top), postcontrast subtraction image (middle), and maximum intensity projection (bottom) show several foci in the right breast. Enhancement kinetics measured from a focus lesion (arrow) demonstrates a rapid initial enhancement followed by washout.

**Figure 3:**
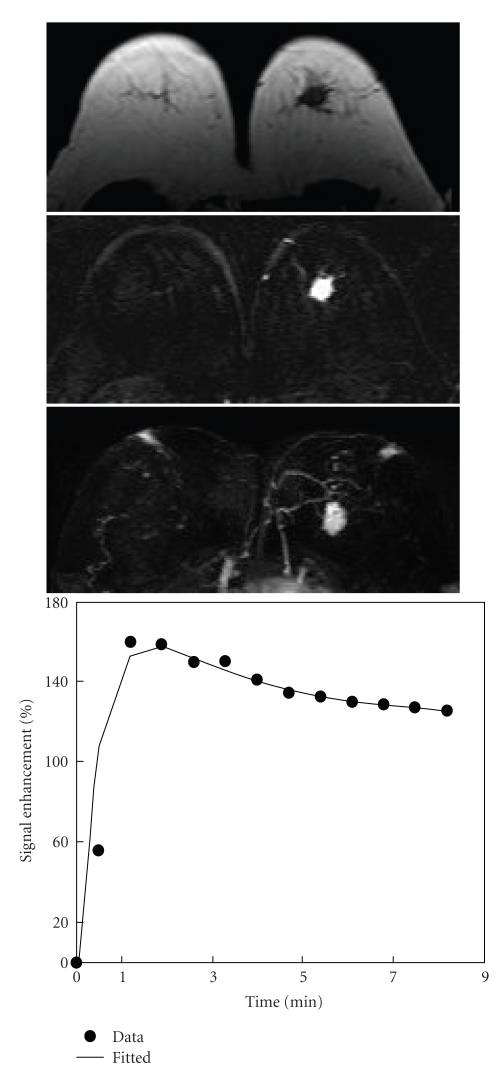
A 61-year-old woman with intermediate-grade pure DCIS. Axial precontrast T1-weighted image (top), postcontrast subtraction image (middle), and maximum intensity projection (bottom) show a mass lesion of 2.2 cm with spiculated margin in the left breast. Enhancement kinetics demonstrates a rapid initial enhancement followed by washout.

**Figure 4:**
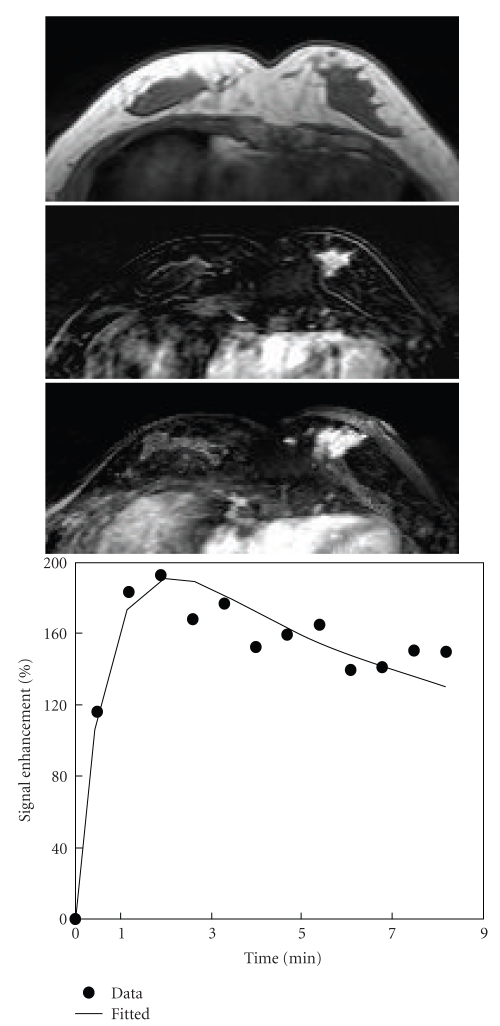
A 39-year-old woman with high-grade pure DCIS. Axial precontrast T1-weighted image (top), postcontrast subtraction image (middle), and maximum intensity projection (bottom) show a 2.8 cm mass lesion with spiculated margin in the left breast. Enhancement kinetics demonstrates a rapid initial enhancement followed by washout.

**Figure 5:**
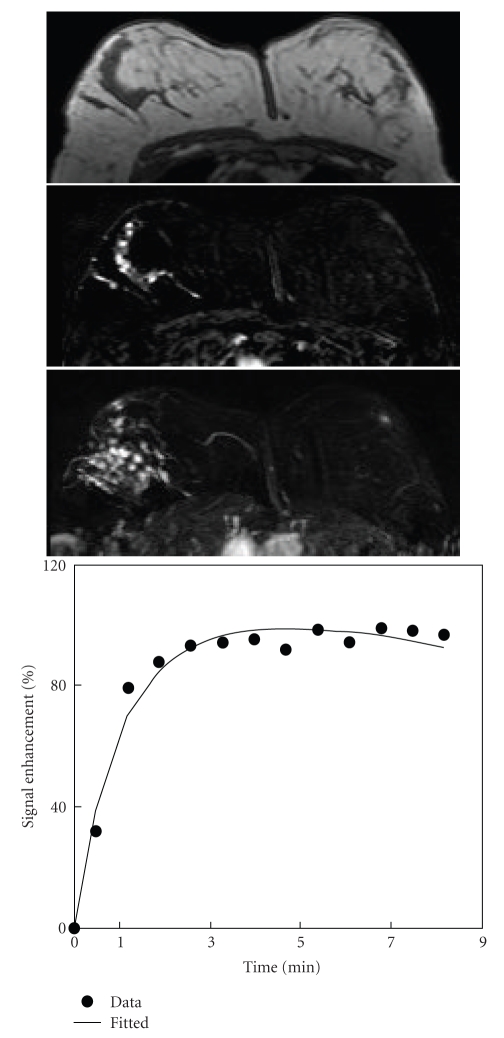
A 47-year-old woman with high-grade pure DCIS. Axial precontrast T1-weighted image (top), postcontrast subtraction image (middle), and maximum intensity projection (bottom) show a Nonmass-like lesion with ductal clumped enhancement pattern in the right breast. Enhancement kinetics demonstrates a rapid initial enhancement then reaching to a plateau.

MR imaging showed contrast-enhanced lesions in 29/31 (94%) cases. Two cases of high-grade DCIS with noncomedo morphology were not detected by MRI and were false negative diagnosis. The imaging features of these 29 cases are summarized in Table 2, and 5 case examples are shown in Figures 1–5. Among these 29 cases, mass lesions were seen in 15 cases (52%) including 12 masses and 3 focus/foci lesions and Nonmass-like lesions in 14 cases (48%). Two cases were multifoci (Figures 2 and 5), and the size was not measured. For the remaining cases, the size ranged from 0.4 to 5 cm with the mean of 1.9 ± 1.2 cm. The mean size was 1.6 ± 1.2 cm for non-high grade DCIS and 2.2 ± 1.3 cm for high-grade DCIS, not significantly different (*P* = .21). 

For the two false negative cases, in mammography, one case was occult, and the other was undetermined (i.e., needed additional imaging evaluation). The mammographically occult case received ultrasound exam and found a suspicious hypoechoic mass in the left breast. After needle biopsy confirming malignancy, the patient received lumpectomy, and a pure DCIS of 1.4 cm was found. The mammographically undetermined case had a palpable nodule in her left breast. This patient was diagnosed with DCIS in the right breast 2 years ago and had already received right mastectomy. She decided to receive left mastectomy, and a pure high grade DCIS of 1.1 cm was found.

#### 3.2.2. Mass Lesions

Among the 15 mass lesions, 9 cases were non-high grade, and 6 were high grade. For the 14 Nonmass-like lesions, 9 lesions were non-high grade, and 5 cases were high grade (*P* = .26). The shape, margin, and internal enhancement patterns were evaluated in 12 mass lesions that were ≥5 mm. The most frequently seen features were irregular shape (50%), spiculated/irregular margin (92%), and heterogeneous enhancement (67%) (Table 3). There was no significant difference in the MR morphology pattern between the non-high-grade and high-grade mass type DCIS lesions. Three case examples are shown in Figure 1 (low grade), Figure 3 (intermediate grade), and Figure 4 (high grade).

#### 3.2.3. Nonmass-Like Lesions

Of 14 Nonmass lesions, 4 lesions showed regional enhancement (28%), 3 showed ductal (22%), and 3 showed focal (22%) enhancements. Figure 2 shows one case example with multiple foci (intermediate grade). Internal enhancement pattern was dominated by the clumped pattern (Figure 5, high grade) (9/14, 64%) followed by the heterogeneous enhancement pattern (4/14, 29%) (Table 4).

### 3.3. MR Enhancement Kinetic Curve Assessment

The enhancement kinetic curves were measured from 27 lesions. Two Grade-II lesions had severe motion artifacts due to patient's movement during the delayed phase, and the enhancement kinetic curves could not be reliably measured. The early postcontrast subtraction images had good quality, and the morphological features of these two lesions could be evaluated. Twenty-one lesions (21/27, 78%) showed the suspicious malignant type enhancement kinetics with a rapid initial enhancement followed by plateau (Figures 1 and 5) or washout (Figures 2–4), including 1/2 Grade-I lesion, 12/14 Grade-II lesions, and 8/11 Grade-III lesions. Therefore, no significant differences were found between enhancement kinetics of high-grade and non-high-grade DCIS (*P* = .38). Among the other 8 enhanced lesions that showed benign type enhancement kinetic curves or undetermined kinetic curves, 6 lesions showed malignant morphological features. Therefore, of the total 31 cases, MRI diagnosed 27 as suspicious of malignancy, with the sensitivity of 87% (27/31).

### 3.4. Correlation of MRI and Mammography Findings

Correlation between screening mammogram and MR Imaging was performed in 22 patients whose mammography reports were available. Sixteen lesions (16/22, 73%) were classified as BI-RADS 4 or 5, as “suspicious” or “highly suspicious of malignancy” on mammography. Five of 22 patients were classified as BI-RADS category 1 (normal mammogram) and one patient as BI-RADS category-0 (need additional imaging evaluation). On MRI, four of 5 patients with normal mammogram were correctly diagnosed as suspicious of malignancy, showing malignant type enhancement kinetics (rapid enhancement followed by washout or plateau) and/or the morphological appearance (irregular shape and spiculated/irregular margin).

## 4. Discussion

Mammography may miss DCIS that does not present certain suspicious patterns of microcalcifications, and MRI may have a complementary role in diagnosis of these lesions. Most of the MR imaging studies of DCIS analyzed mixed patient cohort of pure DCIS and DCIS with invasive components. Several studies reported the MR kinetic and morphologic appearance of pure DCIS correlated with histopathology findings [18–25]. In a study by Kuhl et al., only 56% pure DCIS was detected by mammography, while MRI could detect more than 90% [23]. In another study of 33 pure DCIS patients by Vag et al. [24] the sensitivity of mammography and MRI was 64% and 88%, respectively. In this study, we detected 29/31 (94%) enhancing lesions and diagnosed 27/31 (87%) as suspicious of malignancy. Of the 22 patients whose mammography reports were available, the sensitivity of mammography was 74% (16/22). The result was comparable to other published studies [15, 17, 19]. It was found that most false-negative diagnosis of DCIS by MRI appeared to be non-high-grade, noncomedo-type lesions [23, 32, 33]. In this study, we had two false negative cases of high-grade DCIS with noncomedo morphology. One case showed strong background tissue enhancements in both breasts, and the 1.4 cm lesion could not be identified. The other case showed clumped enhancement in the affected breast, and the 1.1 cm DCIS could not be identified. 

Nuclear grade has been consistently associated with poor prognosis and local recurrence in DCIS [26, 34–36], and the combination of nuclear grade and comedo necrosis was reported to correlate with the risk of local recurrence after breast conserving surgery [34, 35]. Kuhl et al. found that high-grade pure DCIS missed by mammography can be diagnosed by MRI alone [23]. The diagnostic yield of MRI for non-high-grade pure DCIS is also significantly higher than mammography [23]. In the series reported by Vag et al. [31], all 12 mammographically occult DCIS lesions (three low grade, four intermediate grade, five high grade) were correctly diagnosed by MRI. In our series, 4 mammographically occult DCIS lesions were detected by MRI. All these 4 lesions were high grade. With the prognostic significance of early detection for high-grade DCIS, this result further strengthens the recommendation of MRI for diagnosis of DCIS. 

Pure DCIS lesions often appear as Nonmass clumped enhancement in a regional, ductal, or linear distribution [16, 18, 21, 22]. Groves et al. [19] reported a high specificity of focal branching enhanced pattern on MRI for DCIS. As the spatial resolution of MRI improves, it is anticipated that the ductal pattern as linear or linear branching may be seen more often, which can be a signature feature on MRI suggesting DCIS. In our study, the typical morphologic appearance of pure DCIS on MRI was a mass- or a Nonmass-like lesion with heterogeneous or clumped enhancement. Although our study showed a higher percentage of mass lesions (52%) (Figures 3 and 4) compared to previous publications [18, 22], the classification of mass and Nonmass lesions is subjective, and this may lead to variations in the determined percentages. In the study by Viehweg et al. [34], of the 48 enhancing pure DCIS lesions, 35 lesions (35/48, 73%) showed either well-defined (*N* = 12) or ill-defined focal mass lesions (*N* = 23). The percentage will change if some ill-defined mass lesions are reclassified as Nonmass lesions. Similar to that of Viehweg et al. [34], we did not find significant differences between morphologic patterns of high-grade and non-high-grade DCIS.

Pure DCIS does not always exhibit the typical malignant washout kinetic curves and could show persistent and plateau curve types [16, 18, 21, 22]. Jansen et al. reported that the peak enhancement ratio at one minute (E1) for pure DCIS was less than invasive cancers and more than benign lesions [22]. Studies on the enhancement kinetic characteristics among the different nuclear grades of pure DCIS did not show consistent results. Some reports have indicated that the kinetic characteristics of low-grade pure DCIS lesions are different from those of intermediate- and high-grade lesions [18, 37], whereas other studies have revealed no difference [22, 34]. We did not find significant difference between the enhancement kinetics of high-grade and non-high-grade groups. 

For the size measurement of DCIS, overall, MRI was more trustworthy compared with mammography. Different local recurrence rates were found between DCIS lesions smaller and larger than 10 mm, thus an accurate size measurement is important [38]. Boetes et al. [39] showed a −8% deviation of MRI measured tumor size relative to histopathologic size, compared with −29% for mammography. Despite that in general the size measurement is more accurate on MRI, false positive findings and tumor size overestimation may occur on MRI. Kumar et al. [40] reported that cases in which MRI overestimated DCIS were mostly non-high-grade and noncomedo-type lesions. The false-positive enhancement in most cases is due to coexistence of benign proliferative processes such as fibrocystic changes or atypical ductal hyperplasia [16, 40]. 

Several limitations existed in our study, including small case number and retrospective case review. Nevertheless, the reported cases were from consecutive patients referred to participate in a breast MRI research study, with diagnosis of pure DCIS; therefore, allowing objective analysis as presented in this study. The research study was designed to analyze the DCE kinetics, so a protocol with a relatively high temporal resolution (42 seconds compared to 2 minutes used in a clinical setting) was chosen. This could only be accomplished by using a compromised spatial resolution, which would inevitably affect the quality of the image. However, since the tumor size of our subjects was relatively large (1.6 ± 1.2 cm for mass lesions and 2.2 ± 1.3 cm for Nonmass lesions), we believe that the relatively low spatial resolution did not affect our findings on the analysis of lesion morphology. The morphological features of the three focal lesions <5 mm were not evaluated. The feature about spiculated margin might be affected by the low spatial resolution, and the percentage of lesions with spiculated margin could be higher than the reported 42% (5/12 cases). Therefore, the cases with spiculated and irregular margin were reported together. With improved spatial resolution and reduced slice thickness, it is possible that more subtle morphological features, such as linear or linear branching rather than regional enhancements, can be revealed for improved diagnosis of DCIS.

## 5. Conclusion

In this retrospective analysis, the majority (94%) of pure DCIS lesions showed contrast enhancement. MRI was more sensitive in detecting pure DCIS compared to mammography, hence reinforcing the role of MRI in detection and preoperative management of pure DCIS. Most lesions (78%) exhibited the suspicious malignant type enhancement kinetics. However, we did not observe any significant difference in MR morphologic or kinetic features between non-high-grade and high-grade pure DCIS. Therefore, our results did not provide strong evidence to support the use of MRI in staging of DCIS for treatment planning.

## Figures and Tables

**Table 1:** Pathological types and grades of 31 pure DCIS lesions.

	Grade I	Grade II	Grade III	Total
Noncomedo type	2	11	6	19
Comedo type	0	5	7	12

Total	2	16	13	**31**

**Table 2:** MR imaging features of 29 pure DCIS lesions detected by MRI.

	Grade I	Grade II	Grade III	Total %
*Morphology *(*N* = 29)				
Focus/Foci (*N* = 3)	0	3	0	3/29 (11%)
Mass (*N* = 12)	1	5	6	12/29 (41%)
Nonmass (*N* = 14)	1	8	5	14/29 (48%)

*Enhancement kinetics *(*N* = 27)				
Fast washout (*N* = 16)	1	8	7	16/27 (59%)
Fast plateau (*N* = 5)	0	4	1	5/27 (19%)
Continuous enhancement (*N* = 6)	1	2	3	6/27 (22%)

**Table 3:** Morphological characteristics of 12 mass lesions.

	Grade I	Grade II	Grade III	Total
*Margin*				
Spiculated	0	4	1	5 (42%)
Irregular	1	1	4	6 (50%)
Smooth	0	0	1	1 (8%)

*Shape*				
Irregular	1	2	3	6 (50%)
Lobular	0	2	1	3 (25%)
Round/Ovoid	0	1	2	3 (25%)

*Internal Enhancement*				
Rim	0	0	1	1 (8%)
Homogenous	0	1	2	3 (25%)
Heterogeneous	1	4	3	8 (67%)

There was no significant difference of the morphology pattern for mass type lesion between the non-high-grade (I + II) and high-grade (III) pure DCIS.

**Table 4:** Morphological characteristics of 14 Nonmass lesions.

	Grade I	Grade II	Grade III	Total
*Enhancement Type*				
Diffuse	0	0	1	1 (7%)
Regional	1	2	1	4 (28%)
Segmental	0	1	1	2 (14%)
Focal	0	3	0	3 (22%)
Ductal	0	1	2	3 (22%)
Linear	0	1	0	1 (7%)

*Enhancement Pattern*				
Punctate/stippled	0	0	1	1 (7%)
Clumped	1	5	3	9 (64%)
Heterogeneous	0	3	1	4 (29%)

There was no significant difference of the morphology pattern for Nonmass type lesion between the non-high grade (I + II) and high grade (III) pure DCIS.

## References

[1] Pinder SE (2010). Ductal carcinoma in situ (DCIS): pathological features, differential diagnosis, prognostic factors and specimen evaluation. *Modern Pathology*.

[2] Frykberg ER, Bland KI (1994). Overview of the biology and management of ductal carcinoma in situ of the breast. *Cancer*.

[3] Erbas B, Provenzano E, Armes J, Gertig D (2006). The natural history of ductal carcinoma in situ of the breast: a review. *Breast Cancer Research and Treatment*.

[4] Ernster VL, Barclay J, Kerlikowske K, Grady D, Henderson IC (1996). Incidence of and treatment for ductal carcinoma in situ of the breast. *Journal of the American Medical Association*.

[5] Pain JA, Ebbs SR, Hern RPA, Lowe S, Bradbeer JW (1992). Assessment of breast cancer size: a comparison of methods. *European Journal of Surgical Oncology*.

[6] Holland R, Hendriks JHCL, Verbeek ALM, Mravunac M, Schuurmans Stekhoven JH (1990). Extent, distribution, and mammographic/histological correlations of breast ductal carcinoma in situ. *Lancet*.

[7] American College of Radiology (2007). Practice guideline for the management of ductal carcinoma in-situ of the breast (DCIS). *Journal of the American College of Surgeons*.

[8] Bijker N, Meijnen P, Peterse JL (2006). Breast-conserving treatment with or without radiotherapy in ductal carcinoma-in-situ: ten-year results of European Organisation for Research and Treatment of cancer randomized phase III trial 10853—a study by the EORTC Breast Cancer Cooperative Group and EORTC Radiotherapy Group. *Journal of Clinical Oncology*.

[9] Silverstein MJ, Lagios MD, Groshen S (1999). The influence of margin width on local control of ductal carcinoma in situ of the breast. *New England Journal of Medicine*.

[10] MacDonald HR, Silverstein MJ, Lee LA (2006). Margin width as the sole determinant of local recurrence after breast conservation in patients with ductal carcinoma in situ of the breast. *American Journal of Surgery*.

[11] Esserman LJ, Hylton N, Yassa L, Barclay J, Frankel S, Sickles E (1999). Utility of magnetic resonance imaging in the management of breast cancer: evidence for improved preoperative staging. *Journal of Clinical Oncology*.

[12] Orel SG, Schnall MD (2001). MR imaging of the breast for the detection, diagnosis, and staging of breast cancer. *Radiology*.

[13] Gundry KR (2005). The application of breast MRI in staging and screening for breast cancer. *Oncology*.

[14] Gilles R, Zafrani B, Guinebretiere J-M (1995). Ductal carcinoma in situ: MR imaging-histopathologic correlation. *Radiology*.

[15] Orel SG, Mendonca MH, Reynolds C, Schnall MD, Solin LJ, Sullivan DC (1997). MR imaging of ductal carcinoma in situ. *Radiology*.

[16] Shiraishi A, Kurosaki Y, Maehara T, Suzuki M, Kurosumi M (2003). Extension of ductal carcinoma in situ: histopathological association with MR imaging and mammography. *Magnetic Resonance in Medical Sciences*.

[17] Schouten van der Velden AP, Boetes C, Bult P, Wobbes T (2006). The value of magnetic resonance imaging in diagnosis and size assessment of in situ and small invasive breast carcinoma. *American Journal of Surgery*.

[18] Neubauer H, Li M, Kuehne-Heid R, Schneider A, Kaiser WA (2003). High grade and non-high grade ductal carcinoma in situ on dynamic MR mammography: characteristic findings for signal increase and morphological pattern of enhancement. *British Journal of Radiology*.

[19] Groves AM, Warren RML, Godward S, Rajan PS (2005). Characterization of pure high-grade DCIS on magnetic resonance imaging using the evolving breast MR lexicon terminology: can it be differentiated from pure invasive disease?. *Magnetic Resonance Imaging*.

[20] Mariano MN, Van Den Bosch MAAJ, Daniel BL (2005). Contrast-enhanced MRI of ductal carcinoma in situ: characteristics of a new intensity-modulated parametric mapping technique correlated with histopathologic findings. *Journal of Magnetic Resonance Imaging*.

[21] Raza S, Vallejo M, Chikarmane SA, Birdwell RL (2008). Pure ductal carcinoma in situ: a range of MRI features. *American Journal of Roentgenology*.

[22] Jansen SA, Newstead GM, Abe H, Shimauchi A, Schmidt RA, Karczmar GS (2007). Pure ductal carcinoma in situ: kinetic and morphologic MR characteristics compared with mammographic appearance and nuclear grade. *Radiology*.

[23] Kuhl CK, Schrading S, Bieling HB (2007). MRI for diagnosis of pure ductal carcinoma in situ: a prospective observational study. *Lancet*.

[24] Vag T, Baltzer PAT, Renz DM (2008). Diagnosis of ductal carcinoma in situ using contrast-enhanced magnetic resonance mammography compared with conventional mammography. *Clinical Imaging*.

[25] Facius M, Renz DM, Neubauer H (2007). Characteristics of ductal carcinoma in situ in magnetic resonance imaging. *Clinical Imaging*.

[26] Solin LJ (2010). Counterview: pre-operative breast MRI (magnetic resonance imaging) is not recommended for all patients with newly diagnosed breast cancer. *Breast*.

[27] Lim HI, Choi JH, Yang J-H (2010). Does pre-operative breast magnetic resonance imaging in addition to mammography and breast ultrasonography change the operative management of breast carcinoma?. *Breast Cancer Research and Treatment*.

[28] Kerlikowske K, Molinaro A, Cha I (2003). Characteristics associated with recurrence among women with ductal carcinoma in situ treated by lumpectomy. *Journal of the National Cancer Institute*.

[29] MacDonald HR, Silverstein MJ, Mabry H (2005). Local control in ductal carcinoma in situ treated by excision alone: incremental benefit of larger margins. *American Journal of Surgery*.

[30] Lagios MD, Silverstein MJ, Recht A, Lagios M (2003). The Lagios experience. *Ductal Carcinoma In-Situ of the Breast*.

[31] Silverstein MJ, Lagios MD, Craig PH (1996). A prognostic index for ductal carcinoma in situ of the breast. *Cancer*.

[32] American College of Radiology (2003). *Breast Imaging Reporting and Data System Atlas (BI-RADS Atlas)*.

[33] Zuiani C, Francescutti GE, Londero V, Zunnui I, Bazzocchi M (2002). Ductal carcinoma in situ: is there a role for MRI?. *Journal of Experimental and Clinical Cancer Research*.

[34] Viehweg P, Lampe D, Buchmann J, Heywang-Köbrunner SH (2000). In situ and minimally invasive breast cancer: morphologic and kinetic features on contrast-enhanced MR imaging. *Magnetic Resonance Materials in Physics, Biology and Medicine*.

[35] Silverstein MJ, Poller DN, Waisman JR (1995). Prognostic classification of breast ductal carcinoma-in-situ. *Lancet*.

[36] Lagios MD, Silverstein MJ (1997). Ductal carcinoma in situ. The success of breast conservation therapy: a shared experience of two single institutional nonrandomized prospective studies. *Surgical Oncology Clinics of North America*.

[37] Oshida K, Nagashima T, Ueda T (2005). Pharmacokinetic analysis of ductal carcinoma in situ of the breast using dynamic MR mammography. *European Radiology*.

[38] Ottesen GL, Graversen HP, Blichert-Toft M, Christensen IJ, Andersen JA (2000). Carcinoma in situ of the female breast. 10 Year follow-up results of a prospective nationwide study. *Breast Cancer Research and Treatment*.

[39] Boetes C, Mus RDM, Holland R (1995). Breast tumors: comparative accuracy of MR imaging relative to mammography and US for demonstrating extent. *Radiology*.

[40] Kumar AS, Chen DF, Au A (2006). Biologic significance of false-positive magnetic resonance imaging enhancement in the setting of ductal carcinoma in situ. *American Journal of Surgery*.

